# Outcome of Repeated Use of Donor Site for Noncultured Epidermal Cellular Grafting in Stable Vitiligo: A Retrospective Study

**DOI:** 10.1155/2019/7623607

**Published:** 2019-11-11

**Authors:** Vasanop Vachiramon, Korn Triyangkulsri, Duangporn Saengwimol, Kumutnart Chanprapaph

**Affiliations:** ^1^Division of Dermatology, Department of Medicine, Faculty of Medicine Ramathibodi Hospital, Mahidol University, Bangkok, Thailand; ^2^Research Center, Faculty of Medicine Ramathibodi Hospital, Mahidol University, Bangkok, Thailand

## Abstract

**Background:**

Noncultured epidermal suspension (NCES) is a surgical technique which employs cellular grafting onto depigmented lesions. However, scarring and dyschromia at the donor site often occurs.

**Objective:**

To assess the outcome of reusing the same donor site in subsequent sessions of NCES procedures.

**Methods:**

Electronic records of vitiligo patients who had undergone two sessions of NCES procedures were retrospectively reviewed. Information on the first and second NCES was retrieved for analyses.

**Results:**

A total of 30 patients (female 19 and male 11) were included. The majority of patients had nonsegmental vitiligo (66.7%). The median donor-to-recipient ratios were 1 : 3 (1 : 1–1 : 20) for the first session and 1 : 3 (1 : 1–1 : 13.5) for the second session (*p*=0.661). The mean melanocyte count was 220.7 ± 65.5 cells/mm^2^ vs. 242.4 ± 55.3 cells/mm^2^ on the first and second sessions, respectively (*p*=0.440). The mean repigmentation rate was 84.2% (±21.1%) and 82.3 (±22.1%) for the first and second NCESs, respectively (*p*=0.645). The frequency of color mismatch and pigment loss were similar between both sessions (*p*=0.706 and *p*=1.000).

**Conclusions:**

Repeated use of donor sites in subsequent NCES sessions gave comparable repigmentation.

## 1. Introduction

Vitiligo is a common pigmentary disorder that occurs in all age groups, genders, and ethnicities. The prevalence of this condition can be as high as 8% depending on the geographic region [1]. The etiology of vitiligo is complex, and treatment generally requires multiple modalities, including medical treatment and phototherapy. A surgical option is usually employed in cases refractory to the standard treatments. Autologous noncultured epidermal suspension (NCES) is a well-established technique for vitiligo surgery which can produce up to a 50% to 100% repigmentation rate and satisfactory color matching. The process involves harvesting a skin graft from a donor site, usually acquired from the gluteal area, to extract keratinocytes and melanocytes. Then, it would be transferred to a prepared vitiliginous skin [2]. Several factors have been shown to influence the outcome of NCES, such as vitiligo type, duration of disease, location of lesion, and the melanocyte count. Textural change or scar formation of the donor area remains one of the drawbacks of this procedure [3]. Thus, reusing the same donor site for subsequent NCESs may lead to inferior results, as the skin quality of the scarred donor area may not be as good as normal skin. The objective of this study was to assess the outcome of reusing the same donor site for subsequent NCES procedures and to evaluate the side effects after first and second donor site harvesting.

## 2. Materials and Methods

**Figure 1:**
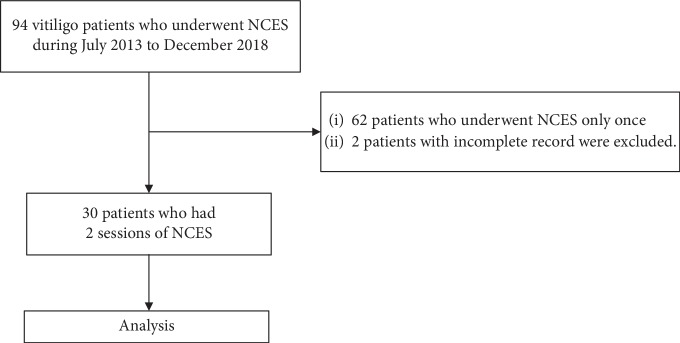
Protocol flow chart. NCES, autologous noncultured epidermal suspension.

This is a retrospective, analytical study assessing the outcome of reusing the same donor site in a second NCES procedure compared with the first NCES procedure in the same patients. The study received an institutional review board approval from the Committee of Human Rights Related to Research Involving Human Subjects, Ramathibodi Hospital, Mahidol University (approval number: ID 12-61-97) (Thai Clinical Trials Registry number: TCTR20190611001). Electronic records of patients diagnosed with vitiligo who underwent NCES between July 1, 2013, and December 31, 2018, were retrieved for research purposes. Thereafter, only patients with complete medical records that had undergone a NCES procedure twice were selected (Figure 1).

### 2.1. NCES Technique

An approximate area of 2.5 × 2.5 cm was marked on the gluteal region. A partial thickness skin graft was acquired from the marked area to obtain an autologous cellular suspension rich in melanocytes. All patients were treated by one expert dermatosurgeon. The skin graft was immersed in a test tube containing 5 mL of normal saline solution and immediately transported to the laboratory. In the laboratory, the skin graft was relocated into a test tube containing 10 mL of phosphate buffer saline (PBS) to remove contaminated fat and blood. The skin graft was then relocated to a test tube containing 10 mL of 0.05% trypsin-ethylenediaminetetraacetic acid (EDTA) solution and incubated at 37°C in the presence of 5% carbon dioxide. After 30 minutes of incubation, the solution was temporarily removed from the incubator, the epidermis was separated from the dermis with fine forceps, and the solution was reincubated for another 30 minutes. After 1 hour of incubation, the skin graft was transferred to a test tube containing 10 mL of fetal bovine serum (FBS) to stop the trypsin-EDTA activity and vortex mixed for 30 seconds. The supernatant was collected and centrifuged for 5 minutes at 1,200 rotations per minute. The supernatant was later removed. The remaining pellet was resuspended again in 10 mL PBS and centrifuged. This process was done twice to ensure adequate removal of trypsin-EDTA and FBS. PBS dilution was performed, and 500 *μ*L of 2.4% hyaluronic acid (hyaluronic acid sodium salt, Merck & Co., Inc., NJ, USA) was added to obtain an appropriate amount of epidermal suspension based on the recipient area. Dye exclusion testing was performed to check the viability of the cells, and the melanocytes were counted using a hemocytometer.

Local anesthesia was achieved on the recipient site. The recipient site was prepared using 10,600 nm carbon dioxide (CO_2_) laser (1000 NAIN, Utech Medical Equipment Co., Ltd, Beijing, China); setting: pulse mode, 2.5–4 watts of power, continuous tissue mode. The epidermal suspension was drawn from the test tube using an automatic pipette and carefully distributed on the prepared recipient site. A dressing was made with Tegaderm (Tegaderm™, 3M Company, St. Paul, MN). The dressing was retained for 7 days and patients were strictly advised to undertake minimal movement on the treated area. Thereafter, the dressing was removed, and the recipient site was subjected to adjuvant phototherapy with narrowband ultraviolet B.

### 2.2. Data Collection

Demographic data including age, gender, and Fitzpatrick's skin type were recorded. Clinical details (e.g., type of vitiligo, age of onset, disease activity, leukotrichia, and associated thyroid diseases) were also recorded. Information on the first and second NCESs retrieved were recorded including the date of operation, donor and recipient sites, NCES technique, donor-to-recipient ratio, cell count, perioperative and postoperative complications, postoperative care, and repigmentation rate. For analytical purposes, the improvement or repigmentation rate was rated as poor (0%–24% repigmentation), fair (25%–50% repigmentation), good (51%–75% repigmentation), very good (76%–90% repigmentation), and excellent (91%–100% repigmentation). Pigmented loss was defined as vitiliginous patches that occurred in an area that has undergone NCES procedure. Pigment loss was categorized as no (0%), partial (<50%), and moderate to complete pigment loss (≥50%).

### 2.3. Statistical Analysis

Categorical data were expressed as frequencies and percentages. Continuous variables were expressed in terms of either mean ± standard deviation for normal distributed data or median (range) for nonnormal distributed data. Data were analyzed using STATA/SE version 14.2 (STATA Corp, College Station, TX). Chi-squared testing, Fisher's exact testing, and marginal homogeneity testing were applied according to categorical data nature. For continuous data, the paired *t*-test was used for normal distributed data and the Wilcoxon sign rank test was used for nonnormal distributed data. A *p* value of <0.05 was considered statistically significant.

## 3. Results

Of the 30 patients included in the study, 19 were female (63.3%) and 11 (36.7%) were male. The mean age was 43.5 (±14.7) years. Most of the patients had nonsegmental vitiligo (NSV) (66.7%) which has been stable for approximately 12 months (1–72 months). Leukotrichia on the recipient site was found in 11 patients (36.7%) (Table 1).

Data regarding the NCES procedures are listed in Table 2. The median lag time between the first and second NCES procedures was 367.5 (49–861) days. The median follow-up was 789 (393–965) days and 424 (265–659) days for 1^st^ and 2^nd^ NCES, respectively. The gluteal region was the only area used as the donor site. Regardless of the size of the recipient area, the donor site was obtained by partial thickness skin graft with a size of 2.5 × 2.5 cm. The median donor-to-recipient ratios were 1 : 3 (1 : 1–1 : 20) for the first session and 1 : 3 (1 : 1–1 : 13.5) for the second session (*p*=0.661). The recipient areas for both NCES sessions were mostly faces and hands (*p*=0.540).

**Figure 2:**
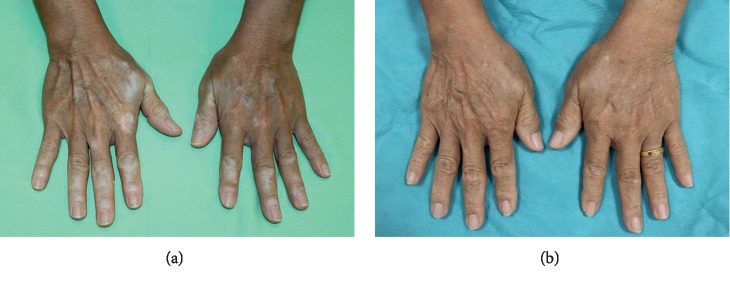
An example of a patient who underwent NCES (a) before the procedure and (b) after the first NCES on the right hand and second NCES on the left hand.

**Figure 3:**
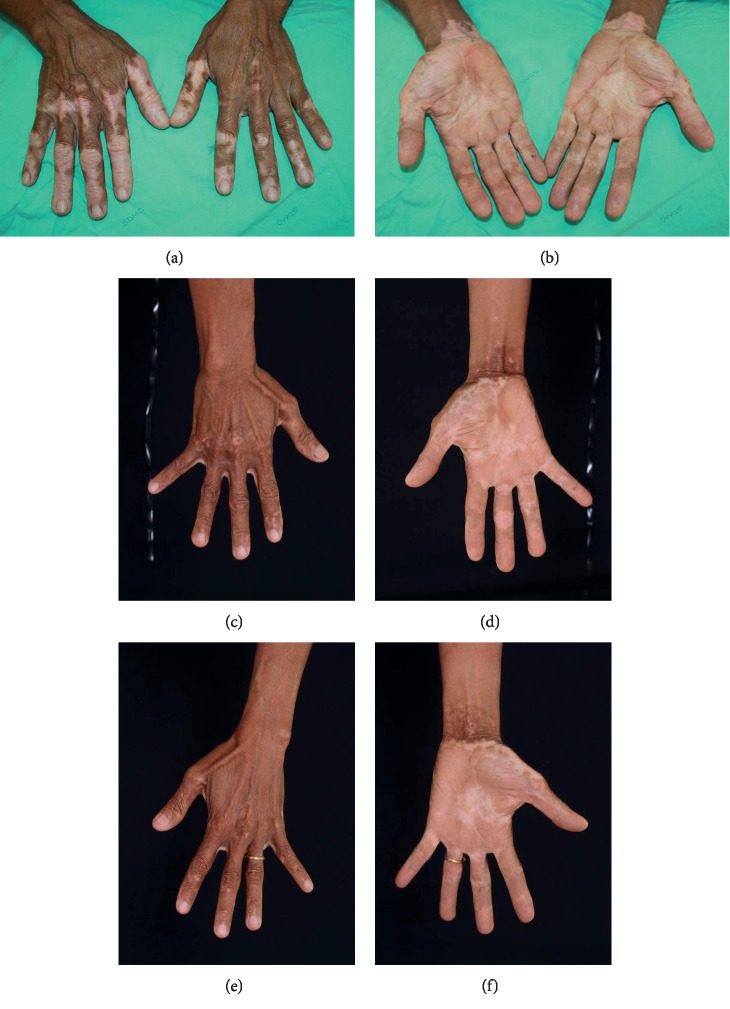
(a, b) Examples of patients who underwent NCES; (c, d) after the first NCES on the right hand; and (e, f) after the second NCES on the left hand.

**Figure 4:**
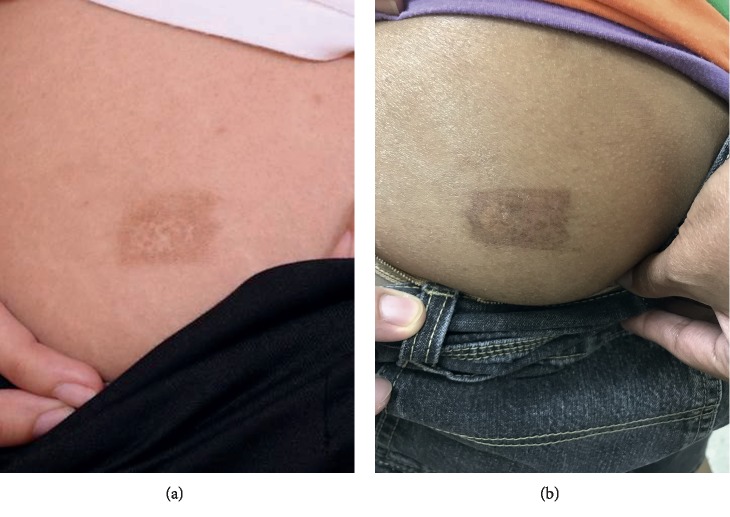
(a, b) Comparison of the cosmetic result at 4 months after procedure on the same donor sites of the first and second NCES.

There was no significant difference in the mean melanocyte counts between the first and second NCESs (220.7 ± 65.5 cells/mm^2^ vs. 242.4 ± 55.3 cells/mm^2^, *p*=0.440). The mean repigmentation rates were 84.2% (±21.1%) and 82.3% (±22.1%) for the first and second NCESs, respectively (*p*=0.645). There was also no difference in the number of patients achieving repigmentations categorized as poor, fair, good, very good, or excellent according to their repigmentation rate (*p*=0.678). Incidences of color mismatch were found in 6 patients (20%) in the first NCES and 7 patients (23.5%) in the second NCES (*p*=0.706). A pigment loss of less than 50% of the treated area occurred equally in 6 patients (20.0%) from each session, and a pigment loss ≥50% of the treated area occurred in 1 patient (3.3%) from each NCES session (*p*=1.000) (Table 2). Figures 2 and 3 demonstrate comparable repigmentation rates for the first and second NCESs. Donor site cosmetic result comparison of the first and second NCESs on the same patient is shown in Figure 4.

With regard to the adverse events, dyspigmentation occurred on the donor site of all patients and appeared as mixed hypo- and hyperpigmentation. Two patients developed hypertrophic scar on the donor site. One patient had hypertrophic scar on the donor site on the first NCES, and another patient developed hypertrophic scar on the second NCES. There was no statistically significant difference in terms of hypertrophic scar formation on the donor site between the first and second sessions (*p*=1.000). One patient who developed hypertrophic scar on the donor site after the first NCES procedure had prolonged bleeding (>5 minutes) from the graft harvesting during the second NCES procedure. Recipient site complications were found only for the second NCES session in which 2 patients developed hypertrophic scars on their hands (*p*=0.157).

## 4. Discussion

Although there has been extensive work to clarify the pathomechanism of vitiligo, there is still currently no cure for vitiligo. In terms of management, general measures such as camouflage and avoidance of koebnerization, including trauma and sunburn, should be recommended to all patients. Current treatment options include topical corticosteroids, topical immunomodulatory agents, phototherapy, photochemotherapy, excimer laser, surgery, and depigmentation of the normal skin. Surgical intervention for vitiligo mainly refers to cellular or tissue grafting performed in stable cases who are refractory to other treatment modalities [4]. Cellular grafting for depigmented lesions was first introduced by Gauthier and Surleve-Bazeille in 1992 [5]. Since then, various technique modifications and upgrades have been developed to improve outcomes and simplify the procedure. Currently, the simplest and most popular cellular grafting technique is the NCES procedure. With an up to 1 : 10 donor-to-recipient ratio, the technique boasts 50% to 100% repigmentation rates and exceptional color matching among most of the treated cases [2, 6–9].

Several factors have been shown to influence the outcome of NCESs and can be categorized into three groups. First are the patient's and disease characteristics. Stability of the disease is the most important factor to determine the success rate of a NCES. Stable segmental vitiligo (SV) appears to have a better treatment response when compared to NSV [3]. Second is the specified technical details of the NCES procedure, including donor site harvesting, donor-to-recipient ratio, laboratory techniques, and recipient site preparation. Third is the postoperative care and postoperative phototherapy [3]. The high success rates of NCES at our center could result from appropriate patient selection (stable disease), a lower donor-to-recipient ratio (median 1 : 3), and routine postoperative phototherapy.

In our study, most of the cases who underwent two sessions of the NCES procedure had NSV and the sites treated were mostly acrofacial regions. NSV patients have relatively large depigmented areas requiring at least two subsequent procedures for complete correction. We included mainly NSV patients into our study. Nevertheless, the outcome of our patients from both treatment sessions was relatively efficacious. Both sessions revealed that over 75% of the patients achieved a very good to excellent response with a mean repigmentation rate of over 80%. Moreover, in two cases, although the donor-to-recipient ratio acquired for the NCES exceeded 1 : 10, they still had very good repigmentation. We emphasize that with careful patient selection and good control of the aforementioned factors, high success rates of NCES can be achieved regardless of the recipient vitiliginous size [10]. Although the incidence of adverse reactions on both the donor and recipient sites of the first and second NCESs did not differ, 1 patient from the second NCES group experienced hypertrophic scar on the donor site. This patient had prolonged bleeding lasting more than 5 minutes during donor skin harvesting on the second NCES procedure. This could have resulted from several injections of triamcinolone on the hypertrophic scar. As the second donor harvesting procedure was performed on a telangiectatic patch secondary to steroid injections, excessive and prolonged bleeding may have occurred.

To the best of our knowledge, our study is the first to demonstrate that using the same donor for the second subsequent NCES procedure does not significantly alter the treatment outcome in terms of repigmentation rate and incidence of color mismatch, as well as the degree of pigment loss. Given that textural change and dyschromia occurred at the donor site in most of the patients, this did not seem to influence the efficacy of the subsequent procedure.

According to a study conducted on a swine model by Travis et al. the numbers of melanocyte were not significantly different in hyperpigmented and hypopigmented hypertrophic scars. However, the difference was the amount of melanin and *α*-melanocyte-stimulating hormone (*α*-MSH) in the lesion [11]. Another study conducted by Alkhalil et al. also demonstrated a difference in cellular activities and gene transcription in hypopigmented and hyperpigmented scars, without differences in the number of melanocytes [12]. In addition, keratinocytes and fibroblasts can be stimulated to produce various cytokines that regulate melanocyte survival, proliferation, migration, and differentiation [13, 14]. The insignificant difference in the outcome of the first and second NCES procedures found in our study could be explained by these findings. We hypothesize that keratinocytes, fibroblasts, and most importantly melanocytes from hypopigmented scars may possibly be “revived” or restimulated after being transferred to a new suitable environment. Further studies are needed to confirm this hypothesis. On the contrary, a higher proportion of patients achieving excellent repigmentation were observed in the second NCES procedure as compared to the first NCES procedure. An explanation for this could be that vitiligo stability is most likely higher as time passes. Therefore, we recommend that the disease should be as stable as possible before performing an NCES procedure. The incidences of color mismatch and stability of the cellular graft were not different between the first and second NCES procedures. These findings confirm the efficacy of repeated use of the donor site. It could be a great option for those who require surgery on a large recipient area and do not wish to have many donor areas following repeated NCES procedure. Regarding the duration between each session of NCES procedure, the earliest time to perform the procedure based on our study was 7 weeks although the wound healing process may take up to 1 year [15]. Autologous noncultured outer root sheath hair follicle cell suspension is a recently introduced technique acquiring anagen hairs extracted from the occipital area and applied as donor. It is another highly effective procedure using a small amount of the donor site. According to a study by Singh et al., vitiligo patients treated with noncultured extracted hair follicle outer root sheath cell suspension showed a comparable efficacy to the NCES procedure [16].

This study may be subject to a few limitations. Due to its retrospective design, some data may have been incomplete and the interval between the first and second NCESs may have varied between individuals. However, as all patients were treated in the same clinic by the same expert dermatosurgeon, and technical nonuniformity is absent. Our data also reflects real life scenarios and the true performance of both the first and second NCESs. Moreover, only the subjective mode of assessment was evaluated. In the future, prospective controlled trials with both subjective and objective modes of assessment may help reassure the results.

## 5. Conclusions

In conclusion, repeated use of skin graft donor sites for a second time in subsequent NCES sessions gave comparable repigmentation rates without increasing significant adverse events.

## Figures and Tables

**Table 1:** Demographic data.

Demographic data	Value
Mean age ± SD; in years	43.5 ± 14.7
Sex (%)	
Male	11 (36.7%)
Female	19 (63.3%)
Skin type (%)	
III	24 (80.0%)
IV	6 (20.0%)
Vitiligo type (%)	
Segmental	9 (30.0%)
Nonsegmental	20 (66.7%)
Unclassified	1 (3.3%)
Mean onset age ± SD in years	32.6 ± 14.2
Median stable duration (range) in months	12 (1–72)
Median duration between 1^st^ and 2^nd^ NCES in days	367.5 (49–861)
Hair involvement (%)	
Yes	11 (36.7%)
No	19 (63.3%)

NCES; autologous noncultured epidermal suspension; SD, standard deviation.

**Table 2:** Data on the first and second NCES sessions.

Data	First NCES	Second NCES	*p* value
Median donor-to-recipient ratio (range)	1 : 3 (1 : 1–1 : 20)	1 : 3 (1 : 1–1 : 13.5)	0.661
Mean melanocyte counts ± SD	220.7 (65.5)	242.4 (55.3)	0.440
Donor complication (%)			
Keloid	1 (3.3%)	1 (3.3%)	1.000
Prolonged bleeding (>5 minutes)	0	1 (3.3%)	0.317
Recipient (%)			0.540
Scalp	1 (3.3%)	0 (0.0%)	
Face	14 (46.7%)	12 (40.0%)	
Neck	2 (6.7%)	1 (3.3%)	
Chest	0 (0.0%)	1 (3.3%)	
Arm(s)	1 (3.3%)	2 (6.7%)	
Hand(s)	11 (36.7%)	13 (43.3%)	
Thigh(s)	1 (3.3%)	0 (0.0%)	
Foot/Feet	0 (0.0%)	1 (3.3%)	
Recipient complication (%)			
Hypertrophic scar	0 (0.0%)	2 (6.7%)	0.157
Mean repigmentation (%) ± SD	84.2 ± 21.1	82.3 ± 22.1	0.645
Patients achieving repigmentation			0.678
0–24% (poor)	1 (3.3%)	2 (6.7%)	
25%–50% (fair)	1 (3.3%)	2 (6.7%)	
51%–75% (good)	3 (10.0%)	3 (10.0%)	
76%–90% (very good)	16 (53.3%)	12 (40.0%)	
91%–100% (excellent)	9 (30%)	11 (36.7%)	
Color mismatch (%)	6 (20.0%)	7 (23.3%)	0.706
Pigment loss (%)			1.000
Partial (<50%)	6 (20.0%)	6 (20.0%)	
≥50%	1 (3.3%)	1 (3.3%)	
No	23 (76.7%)	23 (76.7%)	

NCES; autologous noncultured epidermal suspension; SD, standard deviation.

## Data Availability

The data used to support the findings of this study are available from the corresponding author upon request.
